# Welcome to volume 4 of *Future Science OA*

**DOI:** 10.4155/fsoa-2017-0111

**Published:** 2017-12-07

**Authors:** Francesca Lake

**Affiliations:** 1 Future Science Group, Unitec House, 2 Albert Place, London N3 1QB, UK

**Keywords:** biomedicine, health, medicine, open access, research

I am delighted to introduce the first issue of volume 4 from *Future Science OA*. We are excited to begin our fourth year of publishing research and discussion from across the biomedical-related disciplines, all the way from the bench to the bedside. Although we have some interesting things planned for 2018, in this Foreword, we will take a look back over some highlights from volume 3.

2017 was an excellent year for *Future Science OA*. Our main highlight was the launch of our new-look website, which has allowed us to showcase our articles in an innovative, easy-to-read format, to provide metrics such as downloads and social media mentions for each article and to allow the use of graphical and video abstracts to maximize each article's impact and clarity.

The new website has also allowed us to create topic-specific sections of the journal, allowing our content to be visible to both broad and specific readerships. In addition to common topic areas, such as bioengineering and oncology, we have included a dedicated area for negative research [1]. The *Future Science OA* editorial team believes it is essential that negative and inconclusive research is published in order to ensure transparency and avoid other repeating experiments that have already failed in the past, and we hope to see increasing submissions to this section.

We have also launched the Early Career Research Zone [2]. The *Future Science OA* Young Ambassadors [3] have helped us develop this area, which currently includes career-based interviews, forward-looking perspective pieces written by early career researchers and information regarding the inaugural Future Science Early Career Research Award.

We were particularly proud to launch the Future Science Early Career Research Award in 2017, a scheme to reward an outstanding, biomedical-based, early career researcher. The award saw four finalists chosen from over 30 excellent nominees. The winner was then chosen by our judging panel, which comprised members of our Editorial Board and Young Ambassador Panel, and a vote from over 1200 people. The 2017 winner, Joe Abisambra (University of Kentucky, KY, USA) has now joined our Young Ambassador Panel, and we look forward to him helping us develop more offerings for early career researchers. He also received £1000 in prize winnings and presented a webinar on his Alzheimer's disease research, which you can view at [4]. The Early Career Research Zone is continually evolving, so watch this space!

2017 also saw us accepted for coverage on PubMed Central, which means all of our content is now discoverable via PubMed. This achievement, combined with the excellent quality of our articles and our continued commitment to sharing articles after publication, especially via social media, means that through 2017 we have seen over 11,000 readers per month on average. This is a rise of over 300% from 2016, and has been excellent to see.

In other news, 2017 saw us partner with Publons, a free service for academics that rewards reviewers for their work [5]. We feel that it is important to recognize peer reviewers, and in addition to the discount on publication charges we already provide, our reviewers are now able to add their *Future Science OA* reviews to their Publons profiles.

## Content highlights of 2017

The most-read content through 2017 covered a broad range of topics. The top article, read by over 3500 readers, saw Claus Manniche (Spine Center of Southern Denmark & University of Southern Denmark, Middelfart, Denmark) and Alan Jordan (Broadgate Spine & Joint Clinic, London, UK) discuss how our understanding of Modic changes in back pain has evolved over the past decade [6].

Our 2016 review defining and discussing sarcopenic obesity also features in our most-read list [7]. Boasting a high Altmetrics score of 104, it has had an impressive impact online.

Many articles from our 2017 volume also featured in our most-read list. Of particular note, early career researcher Kevin Deighton (Leeds Beckett University, Leeds, UK) and colleagues produced an excellent forward-looking perspective on the investigation of meaningful effects in physiology research, which was particularly well-read and now has an Altmetric score of 41 [8].

A research article presenting a new method of induced pluripotent stem cell formation avoiding the use of the oncogenes *Myc* and *Lin28* also made an impact, and has an Altmetric score of 49 [9].

Joining these articles in our most-read list are those published in our special issue focusing on organ-on-chip [10]. The issue, featuring guest editor John Greenman (University of Hull, Hull, UK), provided research and review examining microfluidics research, and looked to the future of this exciting field.

## Journal demographics

**Figure 1.**
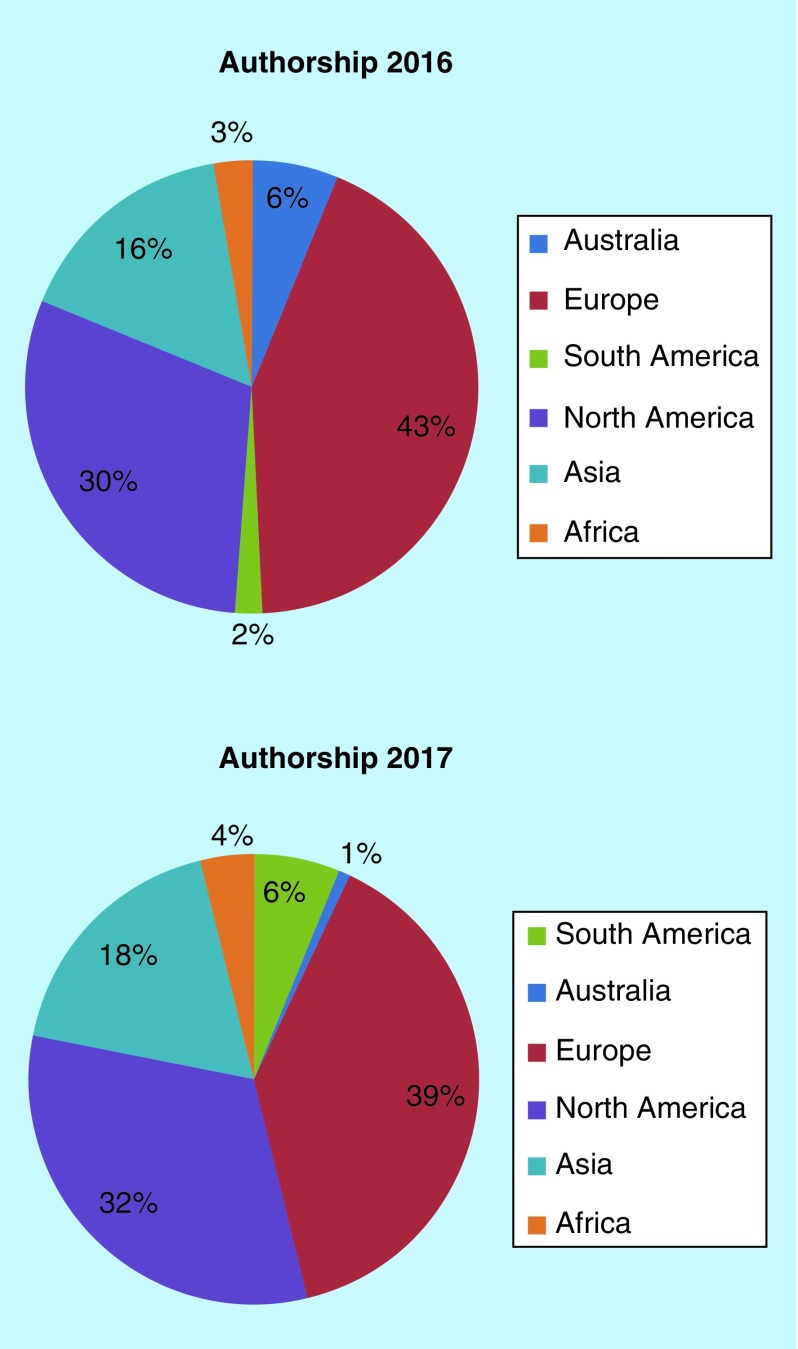
**Authorship demographics through 2016 and 2017 for *Future Science OA*.**

It is always interesting to look back at how our demographics are changing. In 2016, our readers and authors were predominantly from Europe and North America. This trend continues in 2017, with Europe taking the largest portion of the pie, likely owing to the appetite for open access in that geographic region (Figure 1). It is excellent to see a higher proportion of submissions from Africa and South America this year, a trend we hope will continue.

**Figure 2.**
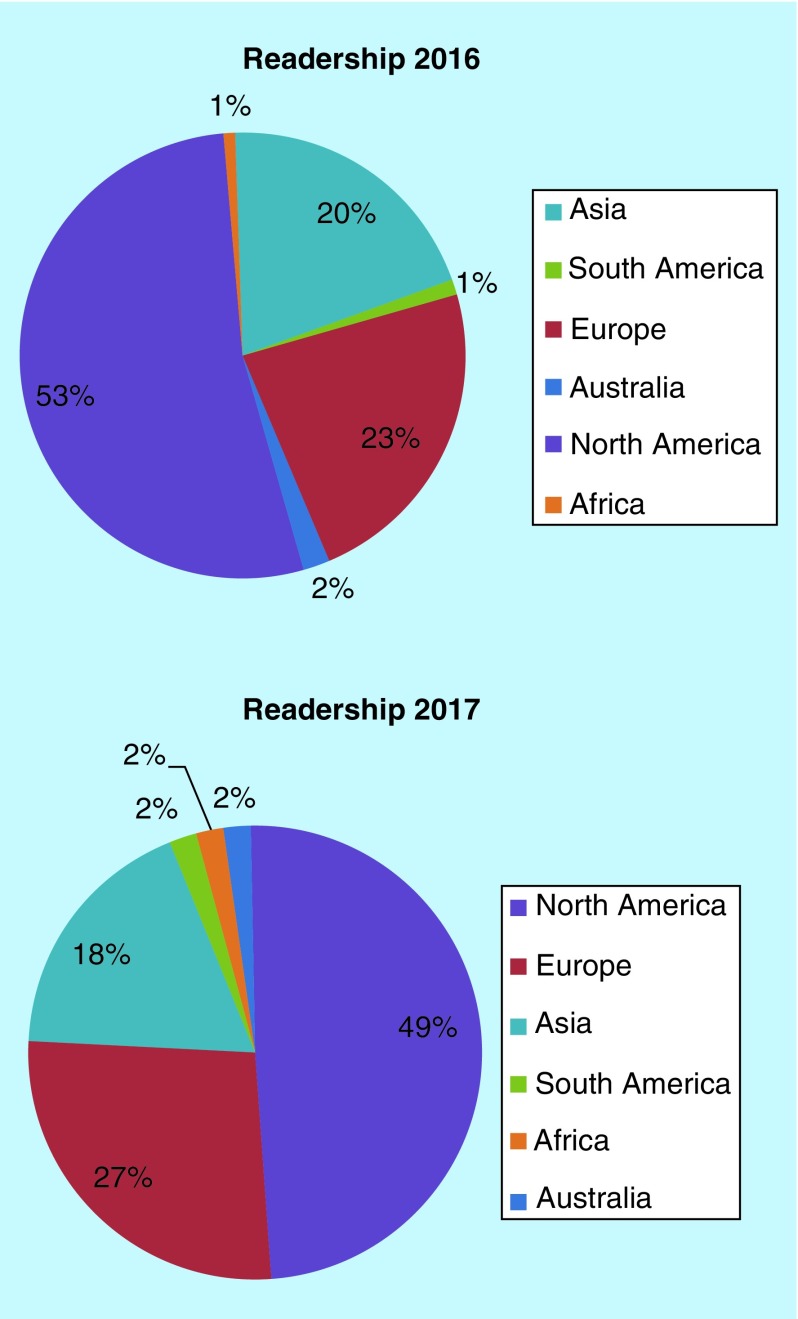
**Readership demographics through 2016 and 2017 for *Future Science OA*.**

In terms of readership, we have seen a huge increase in our readership this year. Our content continues to attract readers from across the globe, which is superb (Figure 2).

## Editorial board

We continue to be hugely grateful to our Editorial Board for their continued support of the journal. This year we have been delighted to introduce a number of new members. Catherine Martel (cardiology; Université de Montréal, Montréal, Canada) was promoted to the board following her excellent work on the Young Ambassador Panel. She has also been joined this year by Matt Gibson (bioengineering; University of Warwick, Warwick, UK), John Greenman (bioengineering; University of Hull), Simon Lo (oncology; University of Washington School of Medicine – Radiation Oncology, WA, USA) and Michael Gold (dermatology; Tennessee Clinical Research Center, Gold Skin Care Center, TN, USA).

## Article outreach

The postpublication dissemination of our articles continues, and we continue to share our publications through our online platforms such as Twitter [11] and Facebook [12]. At the time of writing, our social media efforts have reached around 360,000 users and online sharing accounts for around 2% of our readership. We are excited to see the continued growth in use of innovative ways to discuss scientific research. In support of this, we continue our partnership with Altmetric [13] and Kudos [14], both of which help our authors share their work with the largest possible audience.

## Conclusion

Overall, 2017 has been an excellent year, and we thank our readers, reviewers, authors and Editorial Board members for their continued support. We look forward to working with everyone in 2018.
